# Qualitative assessment of opportunities and challenges to improve evidence-informed health policy-making in Hungary – an EVIPNet situation analysis pilot

**DOI:** 10.1186/s12961-018-0331-z

**Published:** 2018-06-19

**Authors:** Péter Mihalicza, Mark Leys, Ilona Borbás, Szabolcs Szigeti, Olivia Biermann, Tanja Kuchenmüller

**Affiliations:** 1 Semmelweis University Doctoral School, 2 Kőhalom street, Budapest, 1118 Hungary; 2 Vrije Universiteit Brussel, OPIH and EVIPNet Europe steering group, Jette, Belgium; 3 National Healthcare Service Centre, Budapest, Hungary; 4 WHO Country Office for Hungary, Budapest, Hungary; 5 WHO Regional Office for Europe, WHO Secretariat of EVIPNet Europe, Copenhagen, Denmark

**Keywords:** Health policy, Hungary, EVIPNet, Policy-making, Evidence-informed policy-making, Knowledge translation

## Abstract

**Background:**

In evidence-informed policy-making (EIP), major knowledge gaps remain in understanding the context and possibilities for institutionalisation of knowledge translation. In 2014, the WHO Evidence-informed Policy Network (EVIPNet) Europe initiated a number of pilot countries, with Hungary among them, to engage in a ‘situation analysis’ (SA) in order to fill some of those gaps. This contribution discusses the results of the SA in Hungary on research–policy interactions, facilitating factors and potential barriers to establish a knowledge translation platform (KTP).

**Methods:**

In line with the EVIPNet Europe SA Manual, a document analysis, 13 interviews, 3 focus group discussions with 21 participants, and an online survey with 31 respondents were carried out from April to October, 2015. A SA aims to assess the context in which EIP takes form and seeks opportunities to establish a KTP, so information was gathered on the current practice of EIP and knowledge translation, its relevant actors, enablers and barriers for EIP, and opinions on a future KTP. Methodological and researcher triangulation resulted in a narrative synthesis of data, including a comparison with literature. A stakeholder consultation was organised to validate findings.

**Results:**

This study reveals that stakeholders show commitment to produce and use research evidence in Hungarian health policy-making. All stakeholders endorsed the idea of strengthening the systematic use of evidence in decision-making and favoured the idea of establishing a KTP. In line with literature on other countries, some good practices exist on the uptake of evidence in policy-making; however, a systematic approach of developing, translating and using research evidence in health policy processes is lacking. EIP is currently hampered by scattered capacity, coordination problems, high fluctuation in government, an often legalistic and a more ‘symbolic’ rather than practical support for knowledge translation and EIP. The article summarises recommendations on a Hungarian KTP.

**Conclusions:**

Pragmatic adaptation of the SA Manual to local needs proved to be a useful mechanism to provide insight into the Hungarian EIP field and the establishment of a potential KTP. Despite the success of a KTP pilot, it remains unclear how a KTP in Hungary will be institutionalised in a sustainable way.

**Electronic supplementary material:**

The online version of this article (10.1186/s12961-018-0331-z) contains supplementary material, which is available to authorized users.

## Background

Internationally, increased attention is being paid to bridging the gap between health research and policy-making, both at international and local levels [1–4]. Use of research evidence is supportive to health systems strengthening and policy-making, and plays an essential role in improving service delivery [5]. Historically, a shift is being made from ‘narrow’ evidence-based decision-making towards the more pragmatic concept of evidence-informed policy-making (EIP). The latter is used as a terminology to emphasise that evidence (herein meaning research evidence) is only one element within the decision-making process, along with values, habits, resources, experience, pragmatics, lobbyists and judgment [6]. One key element to achieve EIP is knowledge translation (KT) to “*address the gap between what is known from research and implementation of this knowledge by key stakeholders*” [7]. KT needs to backed both by supply-side factors like facilities, resources, skills and system arrangements for generating good-quality analysis and packaging these for policy use, and by demand-side factors including both attitudes and (regulatory) system requirements for using the available evidence (called the institutionalisation of evidence use) [1]. Despite increased awareness for KT, which has enhanced the use of research evidence in health policy-making, a number of key methodological challenges remain, in particular with regard to (1) understanding which KT approaches, tools and mechanisms are most effective, (2) the influence that the context in which KT is implemented plays on research uptake, and (3) how to define and measure the impact of KT [8–10].

In 2005, WHO launched the Evidence-Informed Policy Network (EVIPNet), followed by EVIPNet Europe in the WHO European Region in 2012. EVIPNet promotes partnerships at the country level between policy-makers, researchers and civil society to facilitate both policy development and policy implementation through the use of the best available scientific evidence. Countries become members on a voluntary basis aiming to enhance their EIP and KT capacity. The process of joining EVIPNet Europe holds a transitory phase during which a state (1) conducts country-level activities to prepare to become an EVIPNet Europe member and to establish a country team, and (2) participates in multi-country activities for the purpose of capacity-building. In some cases, the country team is also accompanied by an international consultant hired by the EVIPNet Europe Secretariat. EVIPNet Europe requests its member countries to undertake a ‘situation analysis’ (SA), a description and critical analysis of health policy, health research and practices of EIP in the country to assess the opportunities to establish a knowledge translation platform (KTP). A KTP acts as an institutionalised knowledge broker between the research community and policy-makers (at different levels) to foster research utilisation. It is a facilitating infrastructure which can have a formal, informal, event-specific or thematic organisational form [11]. KTPs are typically multi-disciplinary and develop a credible and legitimate position in policy-making processes, grounded in methodological soundness, transparency and independence from individual stakeholders or policy-makers. A KTP supports and enhances EIP with competencies in problem scoping, evidence gathering, critical appraisal, contextualisation skills, and in active and passive knowledge translation [12]. To ensure sustainability and effectiveness, KTPs need to be adapted to the political, social, research and institutional system of a country and its decision-making mechanisms [8, 13–16]. KTPs differ widely around the world; for example, EVIPNet Peru and EVIPNet Brazil are, for instance, located at the government and municipality level respectively, while EVIPNet Uganda has been successfully based at a university [11]. Other examples of independent institutionalised KTPs exist in Western Europe [17] and many other countries.

Hungary was one of the EVIPNet Europe pilot countries in 2015 [18], among Lithuania, Poland, and Kazakhstan (a previous pilot phase included Slovenia, Moldova, Tajikistan and Kyrgyzstan). Findings of five pilot countries, including Hungary, were presented in 2017 at the European Public Health Conference [19].

The main objective of this contribution is (1) to present and discuss the findings of the SA on policy–research interactions in Hungary and (2) to identify the barriers and opportunities for the establishment of a KTP within the Hungarian context (role, tasks, organisation). This contribution can be a valuable resource for those who seek for concrete manifestations of the approach EVIPNet advertises or wish to study the institutional, procedural, political and policy context of producing, assessing and using research evidence in a post-communist setting.

## Methods

### Data collection

EVIPNet Europe provides a guidance tool [12] to collect data and assess the country situation. This SA Manual guides the systematic and comprehensive identification of important contextual factors that can either support or hinder countries in identifying the organisational and operational niche of the future KTP. It aims to understand the local context by focusing on five major areas, namely (1) the general national context helps to develop a general understanding of the country’s major political, social, public health, socioeconomic and cultural characteristics; (2) the health system and health policy-making context describes the characteristics of its stakeholders, structures, decision-making processes, and key issues in public health and the health system; (3) the health information system elucidates how information on health is gathered, assessed, used and disseminated, and how the system is governed; (4) the context of the national health research system focuses on key research stakeholders, available structures, overall processes and funding mechanisms, as well as key research areas on health in the country; and (5) the existing landscape for EIP providing an overview of current EIP efforts and how they affect the establishment of a new KTP. It recommends the use of document analysis, interviews, focus group discussions and stakeholder consultations to establish and validate findings. For each section, the guide offers prompts or questions to collect information (details of these can be found in the Manual). The SA Manual was developed by experts and critically reviewed, pilot-tested and improved based on the pilot countries’ feedback (including that of Hungary) of its use. To the best of our knowledge, this is the first time that a tool to assess the EIP context of a country has been developed, i.e. there is no available best practice that developers of the Manual could build on.

At the time of the pilot study, the SA Manual was available in a draft version, in English. Since the guidelines are not conceived as a validated ‘research protocol’, but rather as a guidance tool, practical and local context considerations were taken into account. Lessons learned from the first phase of pilots in Slovenia, Moldova, Tajikistan and Kyrgyzstan were shared with the national EVIPNet Europe team (IB, SzSz, PM and an expert from the Ministry of Human Capacities) at multi-country meetings. In the Hungarian pilot, questions in the draft Manual were prioritised by the national team considering local relevance, time constraints and feasibility. Questions that were prioritised at the top were translated to Hungarian. These were then assigned to specific data collection method involving a set of online questionnaires, a focus group guide, an interview guide or a list of questions for the document analysis (see Additional files 1, 2, 3 and 4, respectively). Generally, data on more complex issues and/or opinions were collected by means of focus groups or interviews, while more simple and/or fact-based questions were addressed by questionnaires and document analysis.

Data collection started at the EVIPNet Hungary launch event on April 21, 2015, at the Ministry of Human Capacities and was attended by 32 high-level EIP stakeholders. Participants were introduced to EVIPNet Europe, as well to the purpose and the roadmap for the SA. At the event, a comprehensive stakeholder map (Additional file 5), as well as a preliminary EIP enabler-barrier summary table (Additional file 6), were developed during group work, facilitated by a list of questions (Additional file 7). Both the map and the summary table served as a starting point for further data collection, analysis and validation.

Additional data were collected between April and October 2015 through document analysis, three focus groups (one for researchers, one for policy-makers and one with a mixed audience of all stakeholders), semi-structured interviews and online questionnaires (Table 1). Stakeholders were selected by purposive sampling from research institutes dealing with health systems research (universities and other), government agencies, the Ministry of Human Capacities and various healthcare consultancy and advocacy organisations. Sampling was performed on two brainstorming sessions by the country team and the head of the WHO Country Office in Hungary, based on their judgment and knowledge of relevant stakeholders. We aimed at including high- and mid-level decision-makers, analysts, advisors, academics and representatives of non-governmental organisations (NGOs) in the health policy field with heterogeneous characteristics in order to get as many different perspectives on EIP in health policy as possible. Some of the top-level policy-makers who could not attend the launch event were later reached by means of interviews. Focus groups were facilitated by a professional facilitator using a topic guide. Semi-structured interviews, using a standard set of leading questions, were conducted by PM, IB and SZSZ, an official from the Ministry of Human Capacities and two junior researchers at the National Healthcare Service Center. When formulating opinion on a potential KTP in Hungary, participants were first familiarised with the concept. Additional files 2, 3 and 8 contain the information given at the launch event, at focus group discussions and during interviews, respectively.

**Table 1:** Number and background of participants/respondents for each data collection technique

	Launch event	Focus groups	Semi-structured interviews	Online questionnaires
Researchers	14 (19 invited)	10 (11 invited)	5 (5 invited)	21 (31 sent)
Policy-makers	16 (30 invited)	8 (14 invited)	5 (6 invited)	10 (23 sent)
Other stakeholders (advocacy groups of professionals, patients and providers, clinicians)	2 (4 invited)	3 (3 invited)	3 (3 invited)	0 (0 sent)
Total	32 (53 invited)	21 (28 invited)	13 (14 invited)	31 (54 sent)
Overall response rate	60%	75%	93%	57%
Breakdown by seniority^a^				
Senior level	31	21	13	29
Junior level	1	–	–	2
Breakdown by job focus^b^				
Strategic	13	8	9	8
Operational	19	13	4	23

^a^Senior level: public officials, including heads of divisions and above, public officials with ‘senior advisor’ title, academics including assistant professors and above, leaders of NGOs; Junior level: not senior level

^b^Determined by the research team based on consensus

### Data analysis

The analysis is grounded in a data and methodological triangulation of interviews, focus groups, online questionnaires, the EIP enabler/barrier summary table and a validation meeting.

Focus group discussions, used as the only source of quotes in this manuscript, were transcribed by a professional company using video and audio recordings, and subsequently coded using the ATLAS.ti software by two of the authors (IB, PM) and a junior researcher. Codes were developed inductively using free coding, then classified into themes, which were subsequently clustered according to the SA framework. One country team member (the expert from the Ministry of Human Capacities) compiled an overall summary of semi-structured interviews based on individual interview notes. This synopsis was then reviewed by the other authors to mitigate bias (researcher triangulation). Questionnaire data were processed by MS Excel in the form of simple descriptive statistics.

Data from every source were clustered around the major area’s described in the draft SA and, finally, a narrative synthesis was composed by one of the authors (PM), reviewed and validated by three other members of the EVIPNet Europe team (IB, SzSz and the expert from the Ministry). Based on the narrative synthesis, the authors consensually created a strengths, weaknesses, opportunities, threats (SWOT) table, which emphasised major SWOT of the Hungarian research–policy interface from the point of view of the State Secretariat for Healthcare. The SWOT analysis and participants’ views on a Hungarian KTP were used by the national EVIPNet team to work out – through a series of deliberations – strategies to create a potentially well-functioning KTP within the country’s current institutional context.

Stakeholders could discuss preliminary analyses and the recommendations to establish a KTP on a validation meeting on 5 November, 2015. There were 13 participants with various backgrounds present, all of whom had already engaged in previous activities. However, the final analysis was the sole responsibility of the authors. A summary of the whole data collection, data analysis, synthesis and validation process can be found in Table 2.

**Table 2:** Details on data collection, analysis and synthesis

Data collection phase	Timeline	Type of stakeholders involved	Questions answered	Data analysis method	Overall synthesis method
Launch event	21 April, 2015	High-level EIP stakeholders (top and mid-level policy-makers in office at the time, influential health policy researchers)	Based on a short guidance that were presented to four groups (for details on guidance see Additional file 7)	Qualitative synthesis of the output of each group (see Additional files 5 and 6)	Analysed data were grouped according to the draft SA’s 4 major areas (national context, health policy-making context, health research system, research-policy interface) and a narrative synthesis was produced
Focus group discussions	23 June, 2015 26 June, 2015 29 June, 2015	Mid-level policy-makers, ex top-level policy-makers, influential researchers, representatives of interest groups	Based on a focus group guide (for details see Additional file 2)	Free coding using transcripts, followed by thematic analysis	
Semi-structured interviews	June–August, 2015	Top-level policy-makers in office at the time, leaders of interest groups, influential health policy experts	Based on an interview guide (for details see Additional file 3)	Qualitative synthesis based on interview notes	
Online questionnaires	August–September, 2015	Representatives of divisions of the ministry, government agencies and university departments dealing with health policy research	Based on questionnaires (for details see Additional file 1)	Quantitative questions were summarised in charts and tables, qualitative, free text-based answers were synthetised qualitatively	
Document analysis	August–September, 2015	Not applicable	Based on guiding questions (for details see Additional file 4)	Extraction of relevant information from documents	
Validation meeting	5 November, 2015	Mid-level policy-makers, influential researchers, representative of an NGO	Participants had to reinforce or reject the soundness of main findings in the preliminary report	Qualitative synthesis based on notes	Corrections were made to the preliminary report where necessary

## Results

Although relevant, we did not describe the general country context section, which is normally part of the SA, for reasons of space. We grouped our findings into two core aspects, namely health policy and the interaction between research and policy. This is followed by recommendations from the research team on the establishment of a KTP relying on the SA.

### Health policy field

#### The political context of health policy-making

**Fig. 1:**
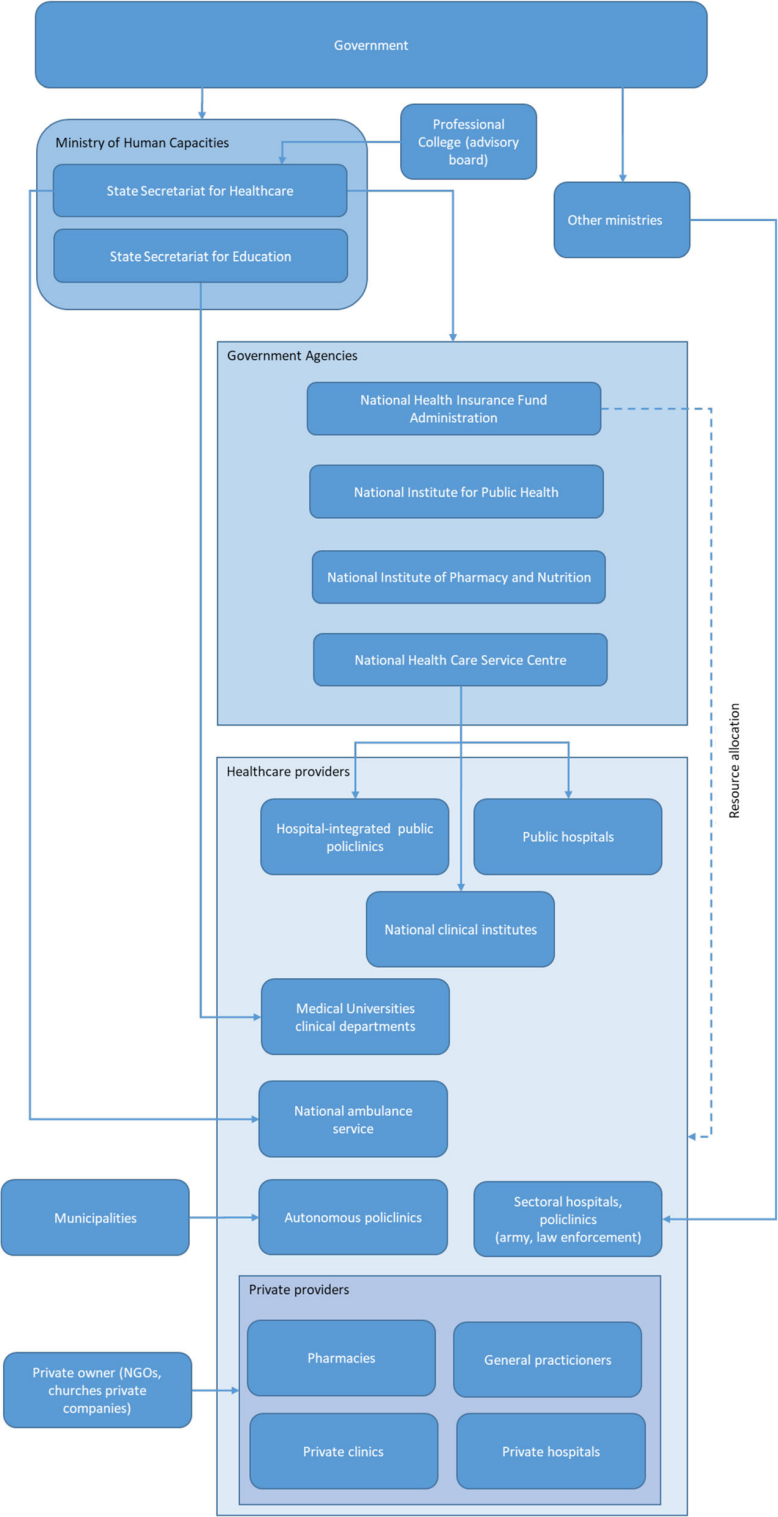
Governance of the Hungarian health system (adapted from [49])

Hungary is a parliamentary democracy. The central government has almost exclusive power to formulate strategic directions and to issue and enforce regulations. National institutions (e.g. ministries, central government agencies) have a major role in the policy cycle (preparing policies as well as decision-making). Local governments have a significantly lower policy-making influence, but are responsible for providing various health services, e.g. primary care (Fig. 1).

The State Secretariat for Healthcare was considered by many as predominant in formulating health policies. However, researchers (many of whom were policy-makers in the past) underline the central supervisory role of the Prime Minister’s Office, the Ministry for National Economy or the government in general. In any case, the roles of actors in the policy-making process depend largely on the topic at stake. There was consensus among the interviewees and focus group participants that lobbyists from the healthcare industry or clinician groups can have substantial influence on certain issues. Many comments were made regarding the (lack of) transparency of decision-making processes, making it difficult to assess how evidence is used. In particular, persons who are not involved in the policy-making process stressed that information is lacking on whether and how much research evidence is used in decisions: “*it is hard to tell how the health decision-making processes happen, as they are not transparent*” (Researcher 1).

As of 2010, health policy was integrated in the Ministry of Human Capacities more particularly, the State Secretariat for Healthcare. This Ministry covers (besides health) several policy domains including education, sports, family affairs, culture, social affairs and religious matters. Some hoped that, through the integration of these policy domains, more collaboration among these sectors would be possible. However, a clear risk emerges that health (system) issues get less direct political attention from the government and the prime minister. Some participants expressed the feeling that healthcare was being marginalised on the policy agenda compared to other issues such as economic growth, competitiveness or the job market. Stakeholders mentioned the lack of a clear delineated policy field for health and healthcare, i.e. there was no predictable financing, no consistency in policy measures derived from strategic directions and no sustainable way of implementing changes. A particular barrier was mentioned by the participants for developing coherent health and healthcare policy, namely that communicating complex health system goals to influential government actors such as the Ministry of National Economy was perceived as a challenge, resulting in a less-than-optimal cooperation on important health issues. Stakeholders felt that targeted knowledge transfer might support awareness raising and understanding about the importance of investments in health and healthcare, thus strengthening budget negotiations. Although examples prove the relevance of intersectoral collaboration (e.g. the Hungarian National Social Inclusion Strategy [20] or the Early Childhood Intervention Programme [21]), it lacks a framework for setting intersectoral health goals and designing or coordinating implementation efforts.

#### Setting strategic objectives in health policy

Explicit health system objectives are framed in laws, strategic documents and regulations, while many implicit objectives can be deduced from government policies. The current strategic orientations evolve mostly around public health issues, primary care, the protection and promotion of patient rights, equal access to services, effectiveness of services and system efficiency. Two major public health strategy documents (Semmelweis Plan [22] and Healthy Hungary Strategy 2014–2020 [23]) line out the directions. According to most participants, however, these documents were only poorly used as practical guidance. Some stakeholders expressed that these strategies could not fulfil their role without operationalised action plans identifying responsibilities and costs. It is challenging, nonetheless, both at national and local levels, to carry out such mid-term planning activities in a coherent and systematic way, if future funds are hard to predict. Besides the abovementioned external factors, many participants assessed that a lack of coordination, adequate communication and trust among stakeholders hinder developing consensus about and implementation of policies. Abrupt organisational changes and rigid modes of operation aggravate this problem.

### The research–policy interface

Building sustainable bridges between the research and policy spheres is at the core of KT. With regards to concrete mechanisms that foster the research–policy interface, more than half of the participating academic institutions responded to the questionnaire that they regularly prepare decision support documents, almost all government agencies stated that they order those at least occasionally, and all said that they prepared policy documents. Respondents of government agencies judged the utilisation of evidence higher than researchers.

However, stakeholders stressed that the often ‘ad hoc’ policy-making process impedes the development and use of research evidence. Thus, providing timely research evidence becomes a key challenge in this environment.

“*Timing of scientific works and policy-making processes, most of the cases, does not concur. Until there isn’t a long term strategy driven decision-making process in place, rather decisions are mostly about quick fixes, we can’t expect to have a work method where if I want to decide on a certain issue, I judge what evidence I need, I get it, evaluate it, and channel it into decision-making*.” (Representative of a government agency 1)

A majority of academic institutions, ministerial departments and government agencies (who filled-in the online questionnaire) expressed high future commitment to support the interface between researchers, providers, advocacy groups and policy-makers. These institutions all indicated a willingness to contribute to develop and coordinate policy programmes, perform data analysis and impact assessments, and organise conferences, discussions and trainings.

Government officials highlighted that a decent inclusive policy development practice was in place in Hungary, because a wide range of stakeholders (including the scientific community) are involved in the review of the planned policy actions through a mandatory-by-law participatory process.

“*…the Decree on public catering was preceded by years of thorough professional preparations; the National Institute for Food and Nutrition Science performed a nutrition survey involving several generations, there have been an extremely widespread professional and civilian consultation and the joint thinking of sectors; in short, this legislation is based on evidence*.” (Policy-maker 1)

Nevertheless, some participants judged the compulsory deliberative process as an ineffective way to incorporate evidence as these consultations generally did not bring tangible outcomes.

“*...the procedural frame is worked out, and we do the analysis, find evidence, based on data and figures, a proposal, then everybody negotiates it and votes here and there. There comes a resolution to support the decision and then, totally independent of* [the previous procedure]*, an opposite decision is made without any explanation. The procedure* [for decision preparation] *is described in detail, the decree is kept, for everybody to see, yet the final decision has nothing to do with the whole thing*.” (Representative of a government agency 2)

Throughout the past 25 years, various initiatives were taken to institutionalise a dialog between research and policy-making (e.g. National Health Council, Regional Health Councils), but none of these proved to be long lasting or influential. Many conferences, roundtables, discussion forums, networks and short courses are organised to disseminate research and raise policy-maker. Participants stressed that, despite the frequent attendance by high-level public sector representatives at these events, policy recommendations and opinions provided were rarely taken into consideration. However, some participants reckoned that a number of individual researchers, universities and certain research institutes were sufficiently influential to impact on policy decisions on a personal and more informal level. In any case, the professional advisory boards of the Ministry can provide a solid base to discuss evidence and advise the policy issues for requests of the Ministry (e.g. advice on new medical technologies). Furthermore, these advisory boards play a role in the clinical governance of medical professions, for instance, by updating clinical guidelines.

In recent years, the government introduced regulations that can foster the use of evidence in policy-making (e.g. compulsory impact assessment, specifying standards for SA and impact assessment of strategic planning exercises, public participation in the formulation of laws and decrees). However, some participants described these regulatory obligations as mainly theoretical or symbolic in nature.

“*Hungary has always been eminent in putting in place things that look good: this is how decision-making should be done. But the problem that these changes stop on the legislative level. So it is not true that we have no frames to fill with content, the problem rather is that there is not too much ambition to really fill it with content.*” (Researcher 2)

“*Every legislative act is accompanied by a so called impact assessment sheet, it’s ridiculous, a mere Excel table that need not even be filled entirely,* [it’s there] *just to have something. No one ever opens it, looks at it, but still looks as if an impact assessment was made about the measure.*” (Representative of a government agency 3)

At the same time, other government officials were more satisfied and did not express a need to reinforce the implementation of the regulations.

“[…] *but what I currently see, that decision-preparation, at least what is done by us, is aiming at a line of conduct based on impact assessment*.” (Representative of a government agency 4)

“*I think that, for instance, talking about the deliberations on Healthy Hungary strategy, or how much dialog we had about smoking regulations or about the tasks of health promotion offices, so I believe that beside particular sectoral interests, there is a will for cooperation too.*” (Policy-maker 1)

Some participants referred to mass media having the role to raise public awareness regarding health policy problems, proposed policy solutions and the rationale for these solutions, thereby putting pressure on policy-makers to enhance the uptake of scientific findings in decision-making. Others thought that the only way to persuade politicians and thus influence politics was through the constituency.

#### Health research, evidence creation and appraisal

The national health research system is shaped mostly by priorities of funders and researchers rather than a central government strategy. In Hungary, the ‘Investment in the Future’ national research and development and innovation strategy 2013–2020, published in 2013 [24], delineates the broad health research goals. However, a complementary guidance by the government for explicit priorities in health systems research does not exist. As a consequence, funding for health (system) research is scattered and is not necessarily connected to policy issues nor aligned with sectoral strategies. The main research funder in Hungary is the European Union, providing Structural and Investment Funds and various research funds (e.g. Framework Programme 7, Horizon 2020). The National Research, Development and Innovation Office provides general research funding via the Hungarian Scientific Research Fund (OTKA) and pharmaceutical companies sponsor drug policy-related research, while WHO dedicates funding to specific health policy research issues through Biennial Collaborative Agreements.

Hungary does not have a government institute whose primary task is to provide policy-relevant research and KT in health system topics like health financing, health system governance, human resources and quality of care. Participants of interviews and focus groups stated that there was potential to enhance EIP as Hungary had sufficient research capacity in academic institutions, as well as research and consultancy companies to support health policy-making. However, public administration lacked the capacity (human resources and knowledge) to access the evidence, synthetise it in the form of high quality decision support tools and apply it in the policy-making process, and was characterised by frequent organisational changes, as participants noted. The Ministry of Human Capacities is particularly overloaded and has high staff-turn-over, inducing lack of time and capacity to engage in preparing strategic decision-making.

“*Hungarian experts have a good reputation, and if we are talking about health economists, Hungary has a great expert lineup on a regional level. There are established research groups, but experts within the government are hurtfully needed. Even those* [research oriented working groups] *that do exist* [within the government] *suffer from considerable fluctuation that makes the situation really difficult.*” (Researcher 3)

The majority of both academic and governmental respondents of online questionnaires mentioned that they have financial resource problems, difficulties in accessing health databases, limited opportunities to participate in international conferences and trainings, and limited access to literature databases.

#### Evidence use

EIP examples were identified in two fields, namely (1) in health technology assessment in the decision-making process of pharmaceuticals [25], and (2) in health system performance assessment [26] for which the first 2-year assessment cycle was completed in 2016. Other good practices mentioned were EU-funded public health projects, where community health promotion methods were grounded in systematic literature reviews, and national policies like the act on the protection of non-smokers [27], the introduction of public health product tax [28, 29], the introduction of the trans-fatty acid decree, the public catering decree, or the human papillomavirus vaccination and screening initiative. Despite these examples, the channelling of evidence into policy-making in Hungary was not always transparent nor systematic, and the quality of the evidence was often not fully assessed, said SA respondents.

### A SA summary and stakeholder opinions on a future KTP

Based on all reflections and commentaries made by stakeholders, a SWOT framework was developed (Table 3). Strengths and weaknesses refer to issues within the authority of the State Secretariat for Healthcare, while opportunities and threats are discussed as those that are outside its authority.

**Table 3:** Strengths, weaknesses, opportunities, threats (SWOT) framework to organise the findings from interviews, focus groups and questionnaires

Strengths • Preparatory documents for policy-making and analysis are very often written • Good practices for evidence-informed policy-making (EIP) in Hungary exist • Many conferences, roundtables, discussion forums, networks, short courses are organised to disseminate research • Main health policy priorities are set in health strategy documents • Almost all academic institutions and government agencies expressed high or medium commitment to support the interface between researchers, healthcare providers, advocacy groups and policy-makers	Weaknesses • Evidence use is not transparent or systematic • Health, healthcare and health system development is not consistently valued as an important policy domain • No systematic follow-up of policy implementation (monitoring and evaluation is not widespread) • Implementation plans are not consistently derived from strategic documents • Lack of coordination, adequate and effective communication among stakeholders • Public administration is lacking the necessary EIP capacity both in terms of human resources as well as EIP knowledge • National health research system is shaped mostly by the priorities of funders and research actors, and not guided by the central government strategically
Opportunities • Law and regulations foster the use of evidence in policy-making and consultative, deliberative processes with stakeholders are in place • Policy-makers express their will and expectations to use scientific evidence • Research capacity to inform health policy-making is available in the country • The country can rely on EU funds and policy-oriented research funds • Legislative framework for creating governmental and sectoral strategies • WHO initiatives to support EIP: the country can build on existing knowledge translation tools, experiences and lessons learned	Threats • Continuation of ad-hoc decisions in health and health system development • A mere ‘symbolic’ commitment to EIP, rather than true support and implementation of knowledge translation and EIP • Uncertainty of available financial resources • Incentives in academic careers do not take into account the support for policy-making • Public administration is strongly bureaucratic and based on laws and regulations, implying a rigid way of operating • Legislative, organisational and policy environment can change rapidly and in an unpredictable manner

#### Opinions on the role of a KTP

Participants endorsed the idea of strengthening EIP and establishing a KTP under the conditions that activities were policy relevant, the KTP maintained its independence and stood for scientific quality. There was general agreement that establishing a KTP would be useful if it aligned with existing activities and capacity in the country. Its role was not to control decision-makers but to supplement the work of the State Secretariat, government agencies and academic institutions, avoiding duplication of activities and unnecessary competition. Its core task would be identifying and channelling of evidence into the policy process. Coordination, synthesis, evidence appraisal and creating platforms of cooperation and discussions should be the core part of the KTP’s tasks, while also providing training in EIP and KT.

#### Location

Numerous participants considered that the State Secretariat for Healthcare could be host of a KTP. At the same time, remarks were made that the daily working culture of the State Secretariat could not provide a protected working time, staff and stable organisational structure for EIP, as its daily routine was mostly guided by quickly changing working priorities. Alternative hosts mentioned were the Professional College (a clinical and health policy advisory body to the State Secretariat for Healthcare), the Hungarian Academy of Science, one of the universities, NGOs or WHO.

#### Formalisation, internal governance and leadership

Many participants stressed that the KTP should be framed in legislation; as a researcher put it: “*what does not have legislative and institutional background does not exist*” (Researcher 4).

Participants considered an independent steering body as part of the organisation’s governance as a necessity. It would consist of representatives of academics, NGOs, advocacy groups and the government. A strong, committed leader who can advocate for the activities of the KTP to politicians and opinion leaders would be vital.

### Recommendations on establishing a KTP in Hungary

The main features of a Hungarian KTP were summarised by the national EVIPNet Europe team. The team adapted the Lavis et al. [17] criteria, descriptions of potential tasks [8, 11, 14–16], SA findings and stakeholder comments during the validation consultation. Initially, three potential KTP institutionalisation scenarios were presented at the latter meeting, of which one was ruled out for feasibility considerations. Details on the two options can be found in Additional file 9.

The core features of a Hungarian KTP

- The Hungarian KTP should be embedded in an existing structure and a network approach should be developed whilst using and channelling existing capacity in the country.
- The KTP should operate sufficiently separate from the ‘regular’ public administration (meaning not being involved in day-to-day, regular tasks of the host organisation), while being close enough to understand policy needs. The KTP should enable policy-makers to express their information needs and these needs should to be handled as priorities by the KTP, with subsequent resource allocation.
- The KTP governance structure should include (1) KTP Board of Trustees, laying down the strategic framework and performing subsequent monitoring of activities; (2) KTP Office with its salaried staff being responsible for the day-to-day operation and coordination; (3) KTP Network of Scientific Advisors as the scientific reflection chamber and which allows access to collaboration with universities, expert groups, NGOs, etc.; (4) the leader of the KTP Office is the main person responsible for carrying out KT tasks and ensuring that the necessary staff is available; (5) ideally, an informal ‘KTP ambassador’ (being a politician or well-known public person committed to the KTP’s goals) who represents the KTP in the media, political happenings and high-level professional forums would be useful, but could be very hard to find.
- Considering the current Hungarian situation, the novelty of the idea of a KTP and the limited resources (both human resources and financial), a gradual implementation strategy would increase the chances of sustainable institutionalisation.
- The Hungarian KTP should cooperate with EVIPNet.

The following basic tasks would be key:

- Support the identification of health policy research priorities and topics in close collaboration with policy-makers and in line with health policy strategies.
- Synthetise and package evidence and communicate context-sensitive policy recommendations to policy-makers in a user-friendly way (evidence briefs).
- Organise policy dialogs and catalyse collaboration among researchers, policy-makers and representatives of health providers and patients.
- Support capacity-building for KT both within and outside the KTP.

## Discussion

This summary of a SA in 2015 in Hungary, one of EVIPNet Europe’s pilot countries, provides a yet unprecedented, systematic insight into the interaction between health research and health policy in Hungary. Emerging insights were developed in dialog with experts from the Hungarian research and policy field. The exercise enables the discussion of opportunities to enhance EIP and KT in Hungary.

This is not a formal research project; rather, the SA tool aims to develop a more systematic rather than just intuitive expert opinion-based approach. An EVIPNet SA aims to gather background information that supports a systematic and comprehensive reflection on the most important local factors that will either support or act as barriers to the establishment and operationalisation of future KTPs. Conducting an SA is an important process to (1) understand the EIP landscape and obtain a baseline; (2) obtain evidence guiding the next steps of the countries KT activities; and (3) to bring various stakeholders together and increasing their awareness on the need to further engage and invest in EIP. While several tools and approaches are meant to assist users in tackling the SA, the Manual is not a rigid protocol. The data collection methods should be, as required, adapted to the local context. The tool aims to create transparency in the sources and data used to assess the local situation. It is clear, as experiences in other EVIPNet pilot countries show, that the use of the SA toolkit is not a ‘formalised protocol’, but requires flexibility, interpretation and continuous coaching, which is also a key feature of qualitative and realist health system research. Moreover, the SA toolkit urges the comparison of the findings with existing literature on the country for their validation. The approaches of other EVIPNet Europe pilot countries in conducting an SA are varied, as every country chose different methodologies from the menu of options presented in the SA Manual.

### Key points of the Hungarian SA in the context of the literature

The findings of this SA are in line with the limited published knowledge on EIP in Hungary [30, 31], papers by the Hungarian Academy of Sciences [32], a paper by a former mid-level government official [33] and a publication relating to employment policy [34]. Erőss et al. [31] discussed examples of how the Hungarian health policy process is not transparent and based on informal mechanisms and informal social networks of experts, politicians and public officials. Szigeti [35, 36] stated that, since the political transformation of the nineties, most policy-makers who focused on possible efficiency gains in the health sector built their strategy on very limited evidence. These findings resonate with ours, in that current policy-making mechanisms give way to opinion-based decision-making through the use of anecdotes on the health system, rather than EIP. Franczel [33] confirmed that individual experts influence decision-making in a non-transparent way. At the same time, Erőss et al. [31] also mirrored our findings on how influential researchers feel ignored within the health policy process, even if they are part of these informal networks. The lack of transparency seems to be an overarching phenomenon that, to the average observer, makes evidence use look even less common than it might be in reality. Even those who are close to the decision-making process at a certain point, or related to a certain issue, do not have a general picture of the whole, and thus have little confidence in the final product. All these findings potentially explain the lack of trust among stakeholders, hampering a systematic and transparent uptake of evidence and research findings.

Orosz [37] explained the relatively low government priority given to public health and health system development from a historic perspective. She stated that, during the communist regime, the Ministry of Health had little influence in deciding on financial resources dedicated to the health system, a system characteristic that pervades after the major political reforms in 1989.

The lack of appropriate planning, consequent implementation and proper monitoring and evaluation of policies, as was mentioned in our SA, was also found by Gajduschek [38], who observed that policies are not grounded in a true analysis of social phenomena, the identification of policy goals is seldom explicit and goals put forward are rarely adequately supported with the means needed for implementation. The mere bureaucratic and regulative approach of governance built on a legalistic paradigm [39] can hamper concrete EIP practices when it is symbolic and formalistic rather than focusing on real change (see also [33, 38] on compulsory impact assessment).

Slovenia is another EVIPNet Europe country that conducted an SA towards the institutionalisation of a KTP [40]. Their assessment has several shared points with the Hungarian case on EIP practices. Relating to compulsory ex ante impact assessment, the Slovenian report talks about a “*gap between intentions and actual practices*” as well as providing several ad hoc examples of successful knowledge translation; however, similarly to us, it found that a systematic, institutionalised approach is lacking. Santoro et al. [41] found the same in their study of 17 Eastern European countries. Petak [42], reporting on the Croatian policy process, mirrored our findings on shortcomings in coordinating government actors and monitoring implementation. Gollust et al. [43] reported that mutual distrust is also key in the United States, while Innvaer et al. [44] noted the same in a systematic review including studies mostly from high-income countries. Shroff et al. [45], covering low- and middle-income countries outside Europe, and Bartlett [46] on countries of Southeast Europe, echoed our participants’ experience that the shortage of adequate skills and high turnover in Ministries of Health are a major obstacle for EIP. Literature often highlights problems on the push side (e.g. communication skills, personal contact, knowledge of the policy process) [47], yet surprisingly, this area did not emerge from our analysis (only one participant mentioned it and no one picked it up). This might be, on the one hand, due to the fact that most of the researchers we asked were or had been policy-makers at some point in their career, and were therefore confident that they indeed know how to properly support policy with evidence. On the other hand, this issue might just simply be ‘under the radar’, as most participants, including most public servants, assume the main problem as being on the pull side.

In summary, we conclude that the Hungarian processes of evidence use are not unique, in the sense that the influence of research evidence on decisions is, at the minimum, uneven in most countries. There are commonalities to be found internationally in specific issues of the policy process and the uptake of evidence.

### Establishing a KTP in Hungary

The SA identified a number of opportunities and barriers for enhancing EIP in the country, and more specifically to create a KTP. Hungarian stakeholders are committed to advance the systematic use of evidence in the policy process and endorsed the idea of establishing a KTP. While, theoretically, six commonly established organisational forms can be distinguished [17], the two Hungarian options aim for an institutionalised organisational configuration with a formal, legal mandate as well as a clear governance structure, budget and staff. Either of the options was considered as a credible support structure to promote research-to-policy processes, at sufficient arms-length from policy-makers and the research community, thus having the necessary credibility and independence to carry out KT mechanisms [48]. Comparable to what is described in the literature, the following key activities need to be performed: (1) priority-setting, harvesting local evidence and synthesise it with global knowledge to provide guidance in policy development and implementation; (2) brokering among stakeholders on key policy issues; (3) packaging syntheses and other communications for specific policy and practice audiences; and (4) strengthening the capacities of researchers, policy-makers and other stakeholders in identifying, accessing and using evidence [14]. However, it should be clear that the establishment and the concrete development of either option will require much further reflection and resources to really establish the KTP.

An eventual standardised comparison of the KTP approaches that different countries took would yield information about the patterns emerging in the EVIPNet pilots. This would require a systematic approach and will be taken up in a future publication.

### Limitations and strengths of the methodology used

This SA exercise has strengths and weaknesses. The EVIPNet Europe draft SA Manual proved to be a supportive tool, but very complex in hindsight. Using it required a balance between (1) the complexity of issues and level of detail needed and (2) the available resources for performing the SA (five persons, of which one junior). Specific issues regarding language and concepts when using an international (English written) Manual needed to be tackled when entering the local field. A considerable amount of time was necessary to customise and contextualise content and methods to local conditions. Additionally, no validation of the Hungarian translations took place, which could distort the distribution of answers and thus the conclusions drawn.

Findings could be subject to a bias due to the method for inclusion of the stakeholders. On the one hand, purposive sampling can ensure, in a time-effective manner, that those who can serve as important primary data sources are included; on the other hand, it can be subject to researcher bias due to errors in researchers’ judgment. As a consequence, this sampling technique can be used to provide insights into the EIP landscape in Hungary and offer various views of a wide range of actors, but is not fit for generalisation of findings to all possible stakeholders. Although the national EVIPNet Europe team reached out to all major stakeholders to the best of their knowledge, researchers were overrepresented compared to other groups, which probably influenced the commentaries. Methodological precautions (triangulation and validation exercises) were performed to reduce interpretation bias. The inductive coding method applied to focus group transcripts allowed for themes to emerge, which contributed to the robustness of results.

PM and IB were public servants at the time of the study, thus having a potential conflict of interest regarding revealing unfavourable data on government processes. We managed this potential conflict by applying rigorous scientific methods (triangulation, peer-review, external validation) and disclosure.

Readers should be aware that, within the pragmatic constraints of resources and time, the methods used do not claim to have made the ultimate assessment of the current Hungarian situation. Future follow-up measurements and in-depth studies will certainly strengthen the future EIP and KT field in Hungary.

### Impact of being involved in EVIPNet activities

The activities of the SA had a wider impact outside the scope of the SA itself – since the submission of the EVIPNet Europe SA to the State Secretariat for Healthcare, the pilot development of an evidence brief for policy (EBP) on antimicrobial resistance had been initiated jointly by WHO Europe and the State Secretariat. This first Hungarian EBP was finalised during 2016–2017 and presented at a policy dialog at the end of 2017. During the course of the EBP development and introduction to stakeholders, one of the KTP options was tested.

## Conclusions

In Hungary, a multi-stakeholder group was formed (including an expert from the Ministry of Health, a staff member of the WHO Country Office, and the two local EIP champions). This combination proved fruitful because (1) the team was well familiarised with EVIPNet Europe’s goals, tools and activities, including the experience of other EVIPNet Europe member countries conducting SAs, and (2) a co-production approach was implemented; both proved beneficial in view of the team understanding the task and moving on swiftly in its implementation. The SA team in Hungary set precedence in applying a diversity of data collection methods to understand the EIP context, exceeding what has been performed in other countries and providing a more refined insight into the existing EIP field.

The SA toolkit proved to be a supportive tool to assess the local health system and health system research context as well as to reflect and engage a discussion on the relevance of EIP and on the potential to establish a KTP. The dialog with stakeholders is a first step to enhance awareness with a wide range of stakeholders at country level. Owing to piloting the SA Manual and process, EVIPNet Europe member countries that are now embarking on conducting a SA benefit from a more user-friendly version of the SA Manual, as well as the possibility of mentoring and peer support provided by the experienced pilot countries.

While one of the proposed KTP options was tested as a pilot, no institutionalised KTP has been created as of 2018. We can conclude that, while a SA can be considered as a useful, or even necessary, step for establishing a KTP, it is not a sufficient one. This Hungarian experience is in line with development in other countries – while a number of them have undertaken SA (Slovenia, Tajikistan, Kazakhstan, Moldova), thus far, no KTP has been established and country teams are still operating in a more informal manner. This is partially due to high government turnovers in the countries that we are operating, institutional changes in the heath policy landscape, the lack of resources to commit to longer-term investment into a KTP and, in some countries, the turnover of experts with whom the EVIPNet Secretariat has worked and trained.

## Additional files

Additional file 1: a Online questionnaire for academic institutes (researchers). b Online questionnaires for government agencies and State Secretariat for Healthcare. (DOCX 169 kb)

Additional file 2: Guide for focus group discussions in ‘Evidence-Informed Policy-making’. (DOCX 29 kb)

Additional file 3: Semi-structured interview questions and information given on the KTP concept. (DOCX 25 kb)

Additional file 4: Document analysis questions. (DOCX 24 kb)

Additional file 5: Stakeholder mapping. Institutions, actors interested in the development of evidence-informed health policy practice and the current experiences – summary of group work at the EVIPNet Hungary launch event. (DOCX 33 kb)

Additional file 6: Enablers and barriers of evidence-informed health policy practice – summary of group work at the EVIPNet Hungary launch event. (DOCX 33 kb)

Additional file 7: Questions supporting group work. Questions supporting group work during EVIPNet Hungary launch event. (DOCX 23 kb)

Additional file 8: Information given on the KTP concept to participants at the EVIPNet Hungary launch event. (DOCX 24 kb)

Additional file 9: Two options for a KTP in Hungary. (DOCX 13 kb)

## Abbreviations

EBP: evidence brief for policy, a policy brief based on the EVIPNet methodology
EIP: evidence-informed policy
EVIPNet: Evidence-Informed Policy Network
KT: knowledge translation
KTP: knowledge translation platform
NGO: non-governmental organisation
SA: situation analysis
SWOT: strengths, weaknesses, opportunities, threats
