# Supplementary material for: Qualitative assessment of opportunities and challenges to improve evidence-informed health policy-making in Hungary – an EVIPNet situation analysis pilot
Source: Health Res Policy Syst. 2018 Jun 19;16:50. doi: 10.1186/s12961-018-0331-z (PMC6006924; doi:10.1186/s12961-018-0331-z)
Supplement: Supplementary file 1 — a Online questionnaire for academic institutes (researchers). b Online questionnaires for government agencies and State Secretariat for Healthcare. (DOCX 169 kb) [file 12961_2018_331_MOESM1_ESM.docx]

Additional file 1.a: Online questionnaire for academic institutes (researchers)

*Since 2015 Hungary is a pilot country of the World Health Organization’s* *Evidence Informed Policy Network (EVIPNet*) European *program. This network aims for promoting the systematic use of health research results and tacit knowledge –as evidence- in health policy decision making.*

*The Hungarian project is coordinated by the AEEK in co-operation with the Ministry of Human Capacities (EMMI). At this stage of the project, we work on Hungarian situation analysis, during which we assess relationships between research, evidence making and health policy decision making, with the institutes, organizations and processes involved.*

*A key feature of the situation analysis is this questionnaire, which we sent to organizations involved in health policy decision making, decision-preparing, knowledge transfer and research. During data process we will not connect any information with the name of the answering person or the concrete organization he/she represents.*

*Answering our questionnaire will take 20-25 minutes. Please give your answers regarding to the institute (university department) you represent.*

*With your contribution you present us valuable information towards the development of a proposal supporting evidence informed decision making and knowledge transfer in healthcare, which we thank you in advance.*

Az űrlap teteje

*Name of the answerer*:

*Please give us the name of the institute (and department, division) in which you are answering our questionnaire.*

**1.** From the list below, please choose up to four activity which are the most typical of your division. Please pick all the valid answers.

- - *Support of preparation for health policy decision and decision making Setting health policy goals.*
  - *Evidence making*
  - *Evaluating quality of evidence.*
  - *Utilization of evidence.*
  - *Financing the making, evaluation of quality or use of evidence.*
  - *Knowledge brokering*
  - *other*

1. Please choose that areas of expertise which are the most typical of your institute.
   - *Clinical research*
   - *Health economy*
   - *Health policy*
   - *Public health*
   - *Health statistics*
   - *Other:…*
2. Does your institute engage in promotion of utilization of evidence (e.g.: researches, impact assessments, analysis) in health policy decision making?
   - *Yes*
   - *No* -> *continue with question 14.*
3. Please sign on a scale of 1 to 3, how often your institute perform the following activity helping the use of evidence in decision making (1: never 2: rarely 3: often).

*Organizing conferences to policy makers, researchers, health care providers and stakeholders*

|  | 1 | 2 | 3 |  |
| --- | --- | --- | --- | --- |
|  |  |  |  |  |

*Helping to build a formal network, working groups, cooperation for policy-makers, stakeholders, health care providers and researchers.*

|  | 1 | 2 | 3 |  |
| --- | --- | --- | --- | --- |
|  |  |  |  |  |

*Organizing policy dialogues for policy-makers, researchers, health care providers and stakeholders.*

|  | 1 | 2 | 3 |  |
| --- | --- | --- | --- | --- |
|  |  |  |  |  |

*Organizing researches, making systematic reviews and decision-preparing studies.*

|  | 1 | 2 | 3 |  |
| --- | --- | --- | --- | --- |
|  |  |  |  |  |

*Ordering researches, systematic reviews and decision-preparing studies.*

|  | 1 | 2 | 3 |  |
| --- | --- | --- | --- | --- |
|  |  |  |  |  |

*Targeted capacity building (education) needed for making a dialogue between policy-makers, health care providers and stakeholders.*

|  | 1 | 2 | 3 |  |
| --- | --- | --- | --- | --- |
|  |  |  |  |  |
|  |  |  |  |  |

*Other*

*If your institute often performs other activities not listed above, helping the use of evidence in health policy processes, please describe them.*

1. Please write down some examples-which your division was involved in the last couple of years (2013-2015) - of successful support of the utilization of evidence in health policy decisions. Please mention what organizations, institutes you co-operated with, and which subject, which roles your partner institute and your division had.

1. How many employees of your institute are engaged in making, evaluating or applying researches as their main task?
2. How do you rate the competence of your institute in making, evaluating or utilizing of researches.
   - *significant*
   - *moderate*
   - *needs to be improved not engaged in such activities*
3. How do you rate the financial resources of your institute in making, evaluating or utilizing of researches.

- *significant*
- *moderate*
- *needs to be improved*
- *not engaged in such activities*

1. How do you rate the competence of your institute in data analysis?
   - *significant*
   - *moderate*
   - *needs to be improved*
   - *not engaged in such activities*

10. How do you rate the competence of your institute in health policy analysis?

- - *significant*
  - *moderate*
  - *needs to be improved*
  - *not engaged in such activities*

11. In your opinion how typical is that your researches, analyses, and impact studies are made for the decision-preparations of health care policies?

|  | 1 |  | 2 | 3 | 4 | 5 |  |
| --- | --- | --- | --- | --- | --- | --- | --- |
|  |  |  |  |  |  |  |  |

12. In your opinion how typical is, that your researches, analyses, and impact studies made for this purpose are utilised during the decision-preparations of health care policies?

|  | 1 | 2 | 3 | 4 | 5 |  |
| --- | --- | --- | --- | --- | --- | --- |
|  |  |  |  |  |  |  |

13. To what extend is your institute engaged in promoting the relation between researchers, health care providers and stakeholders and health policy?

- - *significant*
  - *moderate*
  - *low*

14. If you answered significant or moderate at the last question please explain, what role your institute would like to play and what scope of duties it would like to perform.

15. Does your department have access to all the information and knowledge (including physical and electronic data media in forms of: libraries, subscriptions for journals and databases) needed for your tasks?

- - *Yes -> continue with question 28*
  - *No*

 16. Please describe what kind of shortage your department has in the access of information and knowledge.

Questions that needs subjective evaluation, expert opinion, estimation.

17. In your opinion, how typical is the utilization of evidence (e.g.: researches, impact assessments, analyses) in health policy decision making?

|  | 1 | 2 | 3 | 4 | 5 |  |
| --- | --- | --- | --- | --- | --- | --- |
| *not at all* |  |  |  |  |  | *fully* |

18. In your opinion, on the scale of 1 to 5, to what extend are researches timely available (based on current information, ready on time) for health policy decisions?

| 1 | 2 | 3 | 4 | 5 |  |
| --- | --- | --- | --- | --- | --- |
|  |  |  |  |  |  |

19. In your opinion, on the scale of 1 to 5, to what extend are relevant researches available for health policy decisions?

|  | 1 | 2 | 3 | 4 | 5 |  |
| --- | --- | --- | --- | --- | --- | --- |
|  |  |  |  |  |  |  |

20. In your opinion, on the scale of 1 to 5, to what extend are good quality researches available for health policy decisions?

|  | 1 | 2 | 3 | 4 | 5 |  |
| --- | --- | --- | --- | --- | --- | --- |
|  |  |  |  |  |  |  |
|  |  |  |  |  |  |  |

21. If you know any financial supporter institute, which give resources for the support of evidence use (e.g.: researches, impact assessments, analysis) in health policy processes, please name them.

22.If you know any institutes which give access to systematic reviews online libraries, statistics, indicators or evaluations, in the field of health system, health policy or health economics, focusing on Hungary, please name the institute and the service provided by them.

23. In your opinion, how could the co-operation of researchers, policy makers and professional stakeholders be supported?.

24. If there were a platform with the task of supporting the contact, the day-to-day connection and the continuous information exchange between researchers, policy makers and professional organizations, the availability and utilization of evidence and researches, who would you invite to work for it? Please name concrete institutes or persons if it is possible!

25. In what institutional form, governance structure should this platform work?

26. Please name at least 2 important health policy problems which the platform should start its evidence-synthesizing and knowledge brokering activity targeting health policy decision making processes.

Additional file 1.b: Online questionnaires for government agencies and State Secretariat for Healthcare

*Since 2015 Hungary is a pilot country of the World Health Organization’s* *Evidence Informed Policy Network (EVIPNet* ) *program. This network aims for promoting the systematic use of health research results and functional knowledge –as evidence- in health policy decision making.*

*The Hungarian project is coordinated by the AEEK in co-operation with the Ministry of Human Resources (EMMI). At this stage of the project we work on situation analysis, during which we assess the relationships between research, evidence making and health policy decision making, with the institutes, organizations and processes involved.*

*A key feature of the situation analysis is this questionnaire, which we sent to organizations involved in health policy decision making, decision preparing and research. During data process we will not connect any information with the name of the answering person or the concrete organization he/she represents.*

*Answering our questionnaire will take 20-25 minutes. Please give your answers regard to the institute (to the organizational unit at the State Secretariat for Healthcare) you represent.*

*With your contribution you present us valuable information towards the development of a project amid for helping evidence based decision making and knowledge transfer, which we thank you in advance.*

Az űrlap teteje

*Name of the answerer*:

*Please give us the name of the institute (and division) in which you are answering our questionnaire.*

**

1. *From the list below, please choose up to four activities which are the most typical of your division. Please pick all the valid answers.*

- - *Health policy decision making.*
  - *Preparation of decision making.*
  - *Setting health policy goals.*
  - *Policy tasks (administration, supervision, preparation of laws)*
  - *Evidence making*
  - *Evaluating quality of evidence.*
  - *Utilization of evidence.*
  - *Financing the making, evaluating of quality or use of evidence.*
  - *Knowledge brokering*

2 Please evaluate on a scale 1 to 10 how typical the following statements are about your institute. (1: not typical at all 10: absolutely typical)

*During the institutional decision making process, the operative actions are based on strategic planning.*

*Please sign only one circle in a row*.

|  | 1 | 2 | 3 | 4 | 5 | 6 | 7 | 8 | 9 | 10 |  |
| --- | --- | --- | --- | --- | --- | --- | --- | --- | --- | --- | --- |
|  |  |  |  |  |  |  |  |  |  |  |  |

*Institutional decisions are built on evidence informed proposals, plans.*

|  | 1 | 2 | 3 | 4 | 5 | 6 | 7 | 8 | 9 | 10 |  |
| --- | --- | --- | --- | --- | --- | --- | --- | --- | --- | --- | --- |
|  |  |  |  |  |  |  |  |  |  |  |  |

*During preparation for decision making, the executives of the institute compare the possible outcomes of the alternatives*.

|  | 1 | 2 | 3 | 4 | 5 | 6 | 7 | 8 | 9 | 10 |  |
| --- | --- | --- | --- | --- | --- | --- | --- | --- | --- | --- | --- |
|  |  |  |  |  |  |  |  |  |  |  |  |

*Outcomes of the implementation of decisions are followed up and evaluated.*

|  | 1 | 2 | 3 | 4 | 5 | 6 | 7 | 8 | 9 | 10 |  |
| --- | --- | --- | --- | --- | --- | --- | --- | --- | --- | --- | --- |
|  |  |  |  |  |  |  |  |  |  |  |  |

*Further projects are based upon the results of these evaluations*.

|  | 1 | 2 | 3 | 4 | 5 | 6 | 7 | 8 | 9 | 10 |  |
| --- | --- | --- | --- | --- | --- | --- | --- | --- | --- | --- | --- |
|  |  |  |  |  |  |  |  |  |  |  |  |

1. Does your institute engage in utilization of evidence (e.g.: researches, impact assessments, analyses) in health policy decision making?
   - *Yes*
   - *No* -> *continue with question 18.*
2. Please sign on a scale of 1 to 3, how often your institute apply the following activity helping the use of evidence in decision making (1: never 2: rarely 3: often).

*Organizing conferences to policy makers, researchers, health care providers and stakeholders*

|  | 1 | 2 | 3 |  |
| --- | --- | --- | --- | --- |
|  |  |  |  |  |

*Helping to build a formal network for policy-makers, stakeholders, health care providers and researchers.*

|  | 1 | 2 | 3 |  |
| --- | --- | --- | --- | --- |
|  |  |  |  |  |

*Organizing policy dialogues for policy-makers, researchers, health care providers and stakeholders.*

|  | 1 | 2 | 3 |  |
| --- | --- | --- | --- | --- |
|  |  |  |  |  |

*Organizing research projects, making systematic reviews and decision-preparing studies.*

|  | 1 | 2 | 3 |  |
| --- | --- | --- | --- | --- |
|  |  |  |  |  |

*Ordering researches, systematic reviews and decision-preparing studies.*

|  | 1 | 2 | 3 |  |
| --- | --- | --- | --- | --- |
|  |  |  |  |  |

*Targeted capacity building (education) needed for making a dialogue between policy-makers, health care providers and stakeholders.*

|  | 1 | 2 | 3 |  |
| --- | --- | --- | --- | --- |
|  |  |  |  |  |

*Other*

*If your institute often performs other activities not listed above, helping the use of evidence in health policy processes, please describe them.*

1. Please write down some examples-which your division was involved in the last couple of years (2013-2015) - of successful utilization of evidence in health policy decisions helped by your division. Please mention what organization, institutes you co-operated with, and which subject, which roles your partner institute and your division had.

6. How many employees of your institute are engaged in making, evaluating or applying researches as their main task?*

*Please write down the exact number*

*7. How do you rate the competence of your institute in making, evaluating or applying researches.*

- - *significant*
  - *moderate*
  - *needs to be improved*
  - *not engaged in such activities*

8. How do you rate the financial resources of your institute in making, evaluating or applying researches.

*significant*

- *moderate*
- *needs to be improved*
- *not engaged in such activities*

9. Does your institute have a controlled process which guarantee applying evidence in policy decision making?

- *yes*
- *no*

10. How do you rate the competence of your institute in data analysis?

- - *significant*
  - *moderate*
  - *needs to be improved*
  - *not engaged in such activities*

11. How do you rate the competence of your institute in health policy analysis?

- - *significant*
  - *moderate*
  - *needs to be improved*
  - *not engaged in such activities*

12. To what extend is your institute engaged in promoting the relation between research, health care providers, stakeholders and health policy?

- - *significant*
  - *moderate*
  - *low*

13. If you answered significant or moderate at the last question, please explain, what role your institute would like to play and what scope of duties it would like to perform.

14. Does your department have appropriate access to all the information and knowledge (including physical and electronic data media in forms of: libraries, subscriptions for journals and databases) needed for your tasks?

- - *Yes -> continue with question 28*
  - *No*

15. Please describe what kind of shortages your department has in the access of information and knowledge.

Questions that needs subjective evaluation, expert opinion, estimation.

16. In your opinion, how typical is the utilization of evidence (e.g.: researches, impact assessments, analyses) in health policy decision making?

|  | 1 | 2 | 3 | 4 | 5 |  |
| --- | --- | --- | --- | --- | --- | --- |
|  |  |  |  |  |  |  |

17. In your opinion, on the scale of 1 to 5, to what extend are researches timely available (based on current information, ready on time) for health policy decisions?

|  | 1 | 2 | 3 | 4 | 5 |  |
| --- | --- | --- | --- | --- | --- | --- |
| *not at all* |  |  |  |  |  | *fully* |

18. In your opinion, on the scale of 1 to 5, to what extend are relevant researches available for health policy decisions?

|  | 1 | 2 | 3 | 4 | 5 |  |
| --- | --- | --- | --- | --- | --- | --- |
|  |  |  |  |  |  |  |

19. In your opinion, on the scale of 1 to 5, to what extend are good quality researches available for health policy decisions?

|  | 1 | 2 | 3 | 4 | 5 |  |
| --- | --- | --- | --- | --- | --- | --- |
|  |  |  |  |  |  |  |

20. If you know any financial supporter institutes which give resources for the support of evidence use (e.g.: researches, impact assessments, analyses) in health policy processes, please name them.

21.If you know any institutes which provide access to systematic reviews, online libraries, statistics, indicators or evaluations in the field of health system, health policy or health economics focusing Hungary, please name the institute(s) and the service provided by it (them).

22. In your opinion, how could the co-operation of researchers, policy-makers and professional stakeholders be promoted?

23. If there were a platform with the task of supporting the contact, the day-to-day connection and the continuous information exchange between researchers, policy makers and professional organizations, the availability and utilization of evidence and researches, who would you invite to work for it?

Please, name concrete institutes or persons if it is possible!

  24. In what institutional form, governance structure should this platform work?

25. Please, name at least 2 important health policy problems with which the platform should start its evidence-synthesizing and knowledge- brokering activity targeting health policy decision making processes.
