# Supplementary material for: Qualitative assessment of opportunities and challenges to improve evidence-informed health policy-making in Hungary – an EVIPNet situation analysis pilot
Source: Health Res Policy Syst. 2018 Jun 19;16:50. doi: 10.1186/s12961-018-0331-z (PMC6006924; doi:10.1186/s12961-018-0331-z)
Supplement: Supplementary file 2 — Guide for focus group discussions in ‘Evidence-Informed Policy-making’. (DOCX 29 kb) [file 12961_2018_331_MOESM2_ESM.docx]

Additional file 2: Guide for focus group discussions in ’Evidence-Informed Policy-making’

Introduction to the background of this focus group discussion

- Welcome everybody, my name is [name of the moderator], I am the moderator of this focus group discussion. First, thank you for dignifying us with participating in this discussion. I would like to ask whether everyone sits comfortably (not too hot or cold, dark, strong/weak air conditioning etc.? On the table there are glasses and mineral water for you (beverages, cakes, snacks).
- This discussion, in which you are now involved, is part of a complex research and a more complex project. The whole research is coordinated by the National Healthcare Service Center, commissioned by the Ministry of Human Capacities (EMMI). The research consists of different parts and involves various actors. It includes so-called quantitative and qualitative elements, for example, questionnaires and individual interviews, too. Now you are sitting at the focus group for researchers/health policy.
- I am here as an employee of an external research company, and I am going to fill the role of the focus group’s moderator during the discussion. The whole project, of what this research form a little part, was initiated by the WHO in 2005, certainly worldwide.
- Hungary is a pilot country of the European program of the WHO Evidence Informed Policy Network (EVIPNet) from 2015. The network aims at fostering the systematic use of health research results and practical knowledge in health policy decision-making.
- We are sitting here now within this framework. If I know it properly, many of you participated in the opening ceremony in April (looking around…) Yes, I see that some of you took part and others did not. It is not a problem, it does not affect the participation in the discussion or commenting your opinions either positively, or negatively. You do not need to know any details about the WHO project or the research to take part in the discussion, of course the employees of the ÁEEK could give you information about the operative details after the focus group discussion.
- We make audio and video record of the focus group discussions, which will exclusively be required for the subsequent analysis. Of course, anonymity is guaranteed: nobody’s name will be used in the analysis. /I emphasize that the video record is intended exclusively for research purposes!/
- Please turn your mobile phone off to ensure the smooth conversation, thank you!
- The whole project is organized around predetermined theme groups, and because we are very curious about many things, and because I have a lot of questions, therefore, unfortunately, I will be forced to stop some answers sometimes, take the floor, any interesting idea is told, if it is not closely related to the research topic, OR, when that topic comes up later. Then – if it occurs - I apologize in advance, but as we need to discuss several issues, we have to go along the determined and pre-planned guideline.
- Before we start, I would like to emphasize that everybody’s opinion is important and counts! There are no right or wrong answers – here and now, we are interested only what you would like to say, even if they do not match the opinions of others. I would be glad if it were a conversation/discussion, and I would like to know what you think about the topic. It is important to let everybody speak. Listen to everybody in the group, …and it will be easier for the typewriter if we do not talk simultaneously. ☺
- It is a case in point that a transcription will be made about this discussion, but in the transcription we will change the names in order to guarantee anonymity.
- I would emphasize again that my role here is to ask the pre-planned questions and comply with the times frames. We designed the discussion approximately for 1.5 hours.
- Let’s start slowly. Please, put your name plates, so we all can address each other easily. This is important because during the discussion you can respond to each other’s statements, moreover it is recommended, and you can ask questions to each other relating to the actual topic.

**Opening the floor**: First I would ask everybody to introduce himself/herself very briefly one after another, and say one short sentence about what was on his/her mind when he/she received the invitation, and was coming to this discussion, for example on the street or on the vehicle.

Thank you very much for your introductions, let’s move on to premeditated topics.

**Evidence, Science, Experience, Decision making**

1. To what extent do you think rational, evidence-based approaches, processes form part of health policy decisions? (Can you mention concrete examples for pros and cons?)
2. Who are the most influential persons and determinants of health policy decisions? (Policy-makers, decision supporters, researchers, various stakeholders, etc. knowledge brokers, etc.) Does the concept of „knowledge broker” mean anything for you?
3. In what way could the cooperation of researchers, policy-makers and stakeholders be promoted/facilitated? What kind of stakeholders do exist?
4. Is scientific training for decision-makers important? If it is, how can it be made more effective?
5. What should/could be changed in order to improve capacities, opportunities and efficiency of the interaction between science and decision-making of you or your organization?
6. Which are the main facilitators and barriers related to the changes?
7. How could the governmental advocacy skills of health care be increased? What is the cause of the problem?

**II. Policy decisions and their environment**

1. Do you consider the available capacity for health policy analysis in public sector (ministries, background agencies, universities) and among stakeholders (professional, advocacy organizations, etc.) appropriate (in numbers and quality)?
2. If this capacity is low or inappropriate, are there any needs / resources / practices in any of these groups to charge external researchers?
3. In what way are stakeholders involved in policy making? Is their involvement formal or informal, or are they not involved?
4. Can you see some opportunities for making health policy decision-making processes more transparent? If yes, what kind of opportunities can you see?
5. What kind of relationships between science and decision-makers do you know/are you aware of? Give some examples.
6. What changes are needed to establish the supportive environment for evidence-informed policy (EIP) in Hungary?

**III. KTP**

KTP: Knowledge Translation Platform (making the existing and scientific knowledge useable for policy-makers).

1. Would you consider the creation of KTP useful?
2. What will be the main activities and tasks of KTP? Which themes of policy issues and programs do you think the KTP should start its activity?
3. In your opinion, where the KTP should be hosted? (Whether it should operate as a virtual network, in the Ministry of Human Capacities, in a background institution, at a university etc.)
4. Who, the representatives of which areas would you find appropriate for the membership of the KTP? Please name them personally, if it is possible!
5. How do you see the role of non-governmental/civil sector (industry, patient organizations, professional organizations, etc.) in the future KTP?
6. What main message could be the KTP effectively sold with?
7. What kind of resource and method of resource allocation (global budget, fee-for-research, etc.) can the KTP be operated with?
8. How should the KTP cooperate with other organizations, institutes involved in health care?
9. Which barriers do you see in the establishment of a KTP?
10. In your opinion, what will be the reception of the establishment and operation of the KTP in the political system/ in the media/ among researchers/ in the society as a whole?
11. How and who should manage the KTP?
