# Supplementary material for: Qualitative assessment of opportunities and challenges to improve evidence-informed health policy-making in Hungary – an EVIPNet situation analysis pilot
Source: Health Res Policy Syst. 2018 Jun 19;16:50. doi: 10.1186/s12961-018-0331-z (PMC6006924; doi:10.1186/s12961-018-0331-z)
Supplement: Supplementary file 3 — Semi-structured interview questions and information given on the KTP concept. (DOCX 25 kb) [file 12961_2018_331_MOESM3_ESM.docx]

Additional file 3: Semi-structured Interview questions and information given on the KTP concept

1. To what extent do you think rational, evidence-based approaches, processes form part of health policy decisions? (Can you mention concrete examples for pros and cons?) Where there was evidence, what was the motivation for it?
2. Who are the most influential persons and determinants of health policy decisions (policy-makers, researchers, stakeholders, knowledge brokers, etc.)?
3. By what means/In what way are stakeholders involved in policy making? Is their involvement formal or informal, or are they not involved?
4. How could the governmental advocacy skills of health care be increased? What is the cause of the problem?
5. Do you consider the available capacity for health policy analysis in public sector (ministries, background agencies and universities) and among stakeholders (professional and advocacy organizations, etc.) appropriate (in numbers and quality)?
6. If this capacity is low or inappropriate, are there any needs / resources / practices in any of these groups to charge external researchers?
7. Is scientific training for decision-makers important? If it is, how can it be made more effective?
8. In what way could the cooperation of researchers, policymakers and stakeholders be facilitated/promoted?
9. Translating the previous question to the situation of you or your organization, what should/could be changed in order to improve capacities, opportunities and efficiency of the interaction between science and decision-making of you or your organization?
10. Would you consider the establishment of KTP useful?
11. What would be the main activities and tasks of KTP? Which themes of policy issues and programs do you think the KTP should start its activity?
12. In your opinion, where the KTP should be hosted? (Whether it should operate as a virtual network, in the Ministry of Human Capacities, in a background institution, at an university etc.)
13. Who, the representatives of which areas would you find appropriate for the membership of the KTP? Please name them personally, if it is possible!
14. How do you see the role of non-governmental/civil sector (industry, patient organizations, professional organizations, etc.) in the future KTP?
15. What kind of resource and method of resource allocation (global budget, fee-for-research, etc.) can the KTP be operated with?
16. How should the KTP cooperate with other organizations, institutes involved in health care?
17. How and by whom should the KTP be managed?
18. Which barriers can you see in the establishment of a KTP?
19. In your opinion, what will be the reception of the establishment and operation of the KTP in the political system/ in the media/ among researchers/in the society as a whole?
20. What main message could be formulated for the acceptance of the KTP?

**Information given to interviewees prior to the interview on the KTP concept**

- The Knowledge Translation Platform, as EVIPNet's "engine", serves as a knowledge broker and ensures EVIPNet's strategic goals.
- Depending on the local characteristics, its form, membership and agenda may be different.
- The Knowledge Translation Platform:
  - Encourages the contacts of the actors involved in the process,
  - Supports participants to cooperate in solving problems.
  - Ensures the availability of relevant information for decision-making
  - Develops forums of information transfer to support policy makers and researchers understanding and building mutual trust.
  - Contributes to clarifying the information needs of the policy, to adaptation of general knowledge to local needs.
