# Supplementary material for: Qualitative assessment of opportunities and challenges to improve evidence-informed health policy-making in Hungary – an EVIPNet situation analysis pilot
Source: Health Res Policy Syst. 2018 Jun 19;16:50. doi: 10.1186/s12961-018-0331-z (PMC6006924; doi:10.1186/s12961-018-0331-z)
Supplement: Supplementary file 4 — Document analysis questions. (DOCX 24 kb) [file 12961_2018_331_MOESM4_ESM.docx]

Additional file 4. Document analysis questions

1. Is there an explicitly stated vision, mission and goals for the national health research system?
2. Does a list of explicit national health system research priorities exist?
3. Are there existing national laws, regulations, policies or guidelines on ethical conduct of human subject research and other related areas in the country?
4. What national actors are involved in monitoring and evaluation (M and E) of HSR?
5. What are the key funders and funding agencies of health system research?
6. What are the total public funds allocated to explicit priority health research areas?
7. Total public funding spent on health systems research and total public funding spent on research capacity building
8. Which fields of research are mainly funded (e.g. (bio)medical, health economics/social sciences, health systems, health policy, public health, disease specific etc.)?
9. What is the total number of organizations or departments involved in research?
10. What type of training and education programs on health research are currently offered in the country and what areas are covered (i.e. health systems domain; public health, research methods health policy)?
11. What is the total number of graduates in public health related domains?
12. What is the total number of health system researchers (universities, public institutions, outside the country, NGO, think tank, etc)
13. What is the involvement/participation of patients and the general public in the decision-making process? (strongly involved, moderately, poorly, not involved)
14. Are there national guidelines for the provision of health treatments/services available?
15. To what extent are international guidelines being implemented?
16. What evidence-based medicine and decision- making support tools are used regularly? (HTA, cost-effectiveness analysis, HSPA, other)?
17. Are there any tools for the accountability of the health system actors (public and private, providers, payers, producers of other resources, stewards)?
18. Is health considered a cross-sectoral topic?
19. Are evaluations of patient satisfaction being performed?
20. Are there real opportunities to present and openly discuss research data in local, national and international communities? To what extent do research outcomes become available in the public domain?
21. To what extend is EIP valued and regarded as important within the research community?
