# Supplementary material for: Qualitative assessment of opportunities and challenges to improve evidence-informed health policy-making in Hungary – an EVIPNet situation analysis pilot
Source: Health Res Policy Syst. 2018 Jun 19;16:50. doi: 10.1186/s12961-018-0331-z (PMC6006924; doi:10.1186/s12961-018-0331-z)
Supplement: Supplementary file 5 — Stakeholder mapping. Institutions, actors interested in the development of evidence-informed health policy practice and the current experiences – summary of group work at the EVIPNet Hungary launch event. (DOCX 33 kb) [file 12961_2018_331_MOESM5_ESM.docx]

Additional file 5. Institutions, actors interested in the development of evidence-informed health policy practice and the current experiences – summary of group work at the EVIPNet Hungary launch event

| **Roles** | **Decision-making** | **Decision support** | **Evidence evaluation** | **Evidence generation** | **Data source** | **Implementation** | **Financial support** | **Knowledge brokering** |
| --- | --- | --- | --- | --- | --- | --- | --- | --- |
| Parliament | x |  |  |  |  |  |  |  |
| Government | x |  |  |  |  |  |  |  |
| Ministry of Human Capacities | x |  |  |  |  |  |  |  |
| Ministry for National Economy | x |  |  |  |  |  | x |  |
| Other ministries | x |  |  |  |  |  |  |  |
| National Health Insurance Fund | x | x | x | o | x | x |  |  |
| Office of the Chief Medical Officer | x | x | x | o | x | x |  |  |
| National Institute of Pharmacy and Nutrition | x | x | x | o | x |  |  |  |
| National Healthcare Service Center | x | x | x | o | x |  |  |  |
| Other background institutions |  | x | x |  | x |  |  | x |
| Researchers (e.g. Semmelweis University of Medicine, Corvinus University of Budapest, Eötvös Loránd University, University of Pécs, University of Debrecen, University of Szeged, University of Miskolc, Hungarian Central Statistical Office - Hungarian Demographic Research Institute, Hungarian Academy of Sciences) |  |  | x | x |  |  |  | x |
| Hungarian Academy of Sciences  Hungarian Scientific Research Fund |  |  |  |  |  |  |  |  |
| Professional colleges, Federation of Hungarian Medical Societies |  |  | x |  |  |  |  | x |
| International organizations |  |  | x | x | x |  | x | x |
| Manufacturers, distributors |  |  | x | x |  |  |  |  |
| Service providers |  |  |  | x | x | x |  |  |
| Interest groups |  | they take this role partially |  |  |  |  |  |  |
| Patients |  |  |  | x |  | x |  |  |
| Professional journals |  |  |  |  |  |  |  | x |

(The „evidence generating” and the „evidence evaluating” function must be separated. Key issue is to decide where the evidence would be formed. Certain institutions have concurrent functions.)

Signs:

currently takes this role

does not take this role, but it should
