# Supplementary material for: Qualitative assessment of opportunities and challenges to improve evidence-informed health policy-making in Hungary – an EVIPNet situation analysis pilot
Source: Health Res Policy Syst. 2018 Jun 19;16:50. doi: 10.1186/s12961-018-0331-z (PMC6006924; doi:10.1186/s12961-018-0331-z)
Supplement: Supplementary file 6 — Enablers and barriers of evidence-informed health policy practice – summary of group work at the EVIPNet Hungary launch event. (DOCX 33 kb) [file 12961_2018_331_MOESM6_ESM.docx]

Additional file 6: Enablers and barriers of evidence-informed health policy practice – summary of group work at the EVIPNet Hungary launch event

|  | **Enablers** | **Barriers** |
| --- | --- | --- |
| Political | Strong political authority of the government in decision-making  Stable-than-before sectoral leadership (on the levels of State Ministry of Health, OEP/National Health Insurance Fund)  There is a sectoral strategy  Main priorities are set  (public health, primary care development, slenderizing of specialized care) | Health care is not a priority. Changes in health care overarch political cycles.  Weak sectoral advocacy  But it should be operationalized  Consistent commitment would be needed during implementation  Commissioning of research groups would be needed  Very short public administration deadlines  Ad hoc decisions are frequent |
| Cultural |  | Mixed health culture (the health behaviour of the older generation is passive, that of the youngers is active, but not surely based on evidence)  Different interests between decision-makers and representatives of science, economic sectors and lobbyists  The algorithm of decision-making is difficult to follow, evidence-based decision-making is insufficiently required |
| HR | Good quality professionals | Disproportions in the distribution of professionals (there are too many professionals outside the institutions involved in decision-making) |
| Organizational, institutional background | Existing, good functioning elements, organizations, research groups  Diverse institutions:  National Institute of Pharmacy and Nutrition, National Health Insurance Fund of Hungary, National Healthcare Service Center, National Public Health and Medical Officer Service, National Institute for Health Development, National Center for Patients’ Rights and Documentation, universities, research capacity, professional organizations | The good functioning elements are isolated,  Involvement of scientific groups is missing  There is no cooperation and communication between organizations  There are conflicts between the organizations in many cases  The everyday work of institutions is too rigid  Methodological institutions are not stable, rapid, frequent and deep changes |
| Regulation | Comprehensive legislation of the sector | The legislation process is rigid and slow  There is overregulation in several fields, while in other fields there is no regulation  There is no coherence between certain legal documents  Implementation is insufficient  Lack of framework for health policy planning |
| Resources |  | Scarcity of resources + during the economic crisis the healthcare gets in losing position  No dedicated fund for decision-support research, linked to medium-term policy plans  It is difficult to make financial decision-makers understand the long-term social benefits of certain health policy measures |
| IT | Vast data assets are available | Lack of information in some fields (e.g. there are no valid epidemiologic data which hinders the needs-based planning)  The data is not available in real time  The data use has difficulties (lack of database linkages, lack of IT support and feedback, access is not widespread, problems with validity)  Lack of analytic capacity  Data monopolies  Translation of analysis results into decision-making is not solved |
