# Supplementary material for: Qualitative assessment of opportunities and challenges to improve evidence-informed health policy-making in Hungary – an EVIPNet situation analysis pilot
Source: Health Res Policy Syst. 2018 Jun 19;16:50. doi: 10.1186/s12961-018-0331-z (PMC6006924; doi:10.1186/s12961-018-0331-z)
Supplement: Supplementary file 7 — Questions supporting group work. Questions supporting group work during EVIPNet Hungary launch event. (DOCX 23 kb) [file 12961_2018_331_MOESM7_ESM.docx]

Additional file 7: Questions supporting group work during EVIPNet Hungary launch event

**Subject 1: Facilitators and barriers of evidence-informed health policy practice**

Supporting questions:

- - What are the political, cultural, institutional, individual, regulatory, structural and financial factors supporting and obstructing the Hungarian implementation of evidence-informed policy-making, including the current practice of evidence use?
  - Which strategies could strengthen the existing facilitators and weaken the barriers?
  - What kind of already existing processes - related to research or decision making - have to be considered during introduction of organizational platform supporting evidence use?

**Subject 2. Institutions, actors interested in the development of evidence-informed health policy practice and the current experiences**

Supporting questions:

- - Who are the main actors, stakeholders in the channeling of evidence to policy decision-making?
  - Who should be involved into the processes, who is not participated right now?
  - What roles are taken by stakeholders?
  - What are the positive and negative examples related to actual use of evidence in policy-making?

**Subject 3. Individual roles, expectations about evidence-informed policy-making**

Supporting questions:

- - What do you think about your own role in the development and implementation of processes supporting evidence use?
  - What expectations do you have relating such a system?
