# Supplementary material for: Qualitative assessment of opportunities and challenges to improve evidence-informed health policy-making in Hungary – an EVIPNet situation analysis pilot
Source: Health Res Policy Syst. 2018 Jun 19;16:50. doi: 10.1186/s12961-018-0331-z (PMC6006924; doi:10.1186/s12961-018-0331-z)
Supplement: Supplementary file 8 — Information given on the KTP concept to participants at the EVIPNet Hungary launch event. (DOCX 24 kb) [file 12961_2018_331_MOESM8_ESM.docx]

Additional file 8: Information given on the KTP concept to particpants at the EVIPNet Hungary launch event

**A KTP is a national-level entity designed to bring together the worlds of research, policy and practice.**

- promotes/builds capacity in KT approaches, skills and techniques: e.g. a Rapid Response Service; a policy brief/dialogue; process; clearinghouse; priority setting
- provides a safe space for dialogue and relationships
- brokers and synthesizes
- improve the culture for and practice of research evidence creation, adaptation and use
