# Supplementary material for: Qualitative assessment of opportunities and challenges to improve evidence-informed health policy-making in Hungary – an EVIPNet situation analysis pilot
Source: Health Res Policy Syst. 2018 Jun 19;16:50. doi: 10.1186/s12961-018-0331-z (PMC6006924; doi:10.1186/s12961-018-0331-z)
Supplement: Supplementary file 9 — Two options for a KTP in Hungary. (DOCX 13 kb) [file 12961_2018_331_MOESM9_ESM.docx]

Additional file 9: Two options for a KTP

The **first option** is a mixed organizational and governance model integrating the KTP in the Hungarian Professional College, an advisory body for the State Secretariat for Healthcare regulated by a Ministerial decree. The PC operates with 183 members of 61 medical and paramedical professions forming so called Divisions. Each Division is supported by Councils, whose members are delegated by the professional societies and associations on the field of health care. Its functions and tasks are in line with the new KTP. The KTP Board of Trustees would operate as a Division of the Professional College (PC), while the KTP Office is placed at an already existing government agency. The PC Council would act as the Network of Scientific Advisors.

A distinctive feature is that the KTP Office can directly turn to other Divisions in the PC for consultation, offering a potential for high quality multidisciplinary work.

However, in order to realize the objective of EIP, embedding the KTP in the PC structure, would require changes in the operation, delegation and supervisory structure. More focus needs be put on high quality, operative work at *every* Division which would require additional resources and substantial deliberation efforts.

The **second option** is establishing a methodological institute within an existing government agency, integrating the board of trustees, the KTP office and the network of scientific advisors. This model needs political and management level commitment and trust in the KTP to ensure independence. Compared to the first option this latter offers a more transparent and compact organizational structure with clear rights and responsibilities.
